# Potential Use of MEG to Understand Abnormalities in Auditory Function in Clinical Populations

**DOI:** 10.3389/fnhum.2014.00151

**Published:** 2014-03-13

**Authors:** Eric Larson, Adrian K. C. Lee

**Affiliations:** ^1^Institute for Learning and Brain Sciences, University of Washington, Seattle, WA, USA; ^2^Department of Speech and Hearing Sciences, University of Washington, Seattle, WA, USA

**Keywords:** magnetoencephalography, minimum-norm estimates, audition, clinical practice, central auditory processing disorder

## Abstract

Magnetoencephalography (MEG) provides a direct, non-invasive view of neural activity with millisecond temporal precision. Recent developments in MEG analysis allow for improved source localization and mapping of connectivity between brain regions, expanding the possibilities for using MEG as a diagnostic tool. In this paper, we first describe inverse imaging methods (e.g., minimum-norm estimation) and functional connectivity measures, and how they can provide insights into cortical processing. We then offer a perspective on how these techniques could be used to understand and evaluate auditory pathologies that often manifest during development. Here we focus specifically on how MEG inverse imaging, by providing anatomically based interpretation of neural activity, may allow us to test which aspects of cortical processing play a role in (central) auditory processing disorder [(C)APD]. Appropriately combining auditory paradigms with MEG analysis could eventually prove useful for a hypothesis-driven understanding and diagnosis of (C)APD or other disorders, as well as the evaluation of the effectiveness of intervention strategies.

## Introduction

Magnetoencephalography (MEG) is a non-invasive human physiology technique that records magnetic field patterns from hundreds of sensors simultaneously with millisecond precision. Like electroencephalography (EEG), MEG principally measures neural activity from large populations of (>50,000) cortical pyramidal neurons firing with close temporal and spatial alignment (Okada, 1983; Hämäläinen et al., 1993), but measures the magnetic field surrounding the head as opposed to the electric potential at the scalp. This leads to less distortion of MEG signals due to the propagation of signals through the scull and scalp, and gives MEG a complementary sensitivity profile to neural sources (Goldenholz et al., 2008) and finer spatial resolution (e.g., Sharon et al., 2007), allowing for potentially more accurate mapping of sensor measurements onto neural structures. Combining MEG and EEG for imaging cortical sources has also been shown to sharpen localization compared to using either measurement alone (Sharon et al., 2007), indicating that MEG and EEG provide complementary methods for measuring neural activity.

Magnetoencephalography has been used for over 30 years to better understand auditory processing. About a decade after the first reported MEG recording of human brain activity (Cohen, 1968), researchers used MEG recordings to disambiguate the location of neural generators of the auditory N100 response to stimuli, showing they emerge from bilateral auditory cortices instead of frontal regions (Hari et al., 1980). Since then MEG has seen widespread use in basic science research in audition as well as other domains, accompanied by expansions into sophisticated analysis methods. The high temporal resolution of MEG as an electrophysiological measure positions it to be especially appropriate for use in analyzing auditory processing, where stimuli are necessarily characterized in terms of their fluctuations in time (e.g., see Shamma et al., 2011). Although multiple authors have covered standard practices for conducting MEG studies (Barkley, 2004; Liu et al., 2010; Lee et al., 2012) and reporting the methods and results (Gross et al., 2013) from MEG experiments, adoption of MEG as a tool to facilitate diagnosis has remained limited.

Here we will provide a perspective on potential ways in which MEG could, with additional time, effort, and validation, influence clinical practice for developmental pathology. In terms of methodology, we will specifically focus here on inverse imaging methods because they provide an accurate mapping from sensor readings onto underlying neural anatomical structures. Additionally, connectivity measures derived from these inverse imaging methods can also be informative in identifying interactions between nodes of brain networks. In terms of application, we will focus on how MEG could come to influence practice surrounding (central) auditory processing disorder [(C)APD], which often manifests during childhood as difficulty hearing in noisy environments (e.g., classrooms) despite normal audiometric measurements (i.e., often interpreted as typical peripheral sensitivity). We speculate about how MEG, auditory paradigms, and specific analysis methods could be combined to diagnose or help guide treatment of (C)APD, or, by extension, other disorders that manifest during development.

## MEG Analysis Methods

A common method of MEG analysis is to examine trial-averaged signals directly at the sensor-level, commonly known as event-related fields or potentials (ERF/Ps), which could prove useful for multiple clinical purposes (Reite et al., 1999). Recent reviews have also covered how ERF/Ps can be used to highlight different stages of auditory processing (Alain and Tremblay, 2007; Näätänen et al., 2007) and thus could eventually be used for clinical assessment and therapy (Näätänen, 2003; Duncan et al., 2009). In some situations, however, analyzing MEG or EEG signals in sensor-space can make it difficult to infer which neural structures underlie observed changes, as there may not always be a simple relationship between a particular sensor time series and neuroanatomical function. To link sensor readings to neurophysiology, clinical MEG use up to the present day has primarily relied upon equivalent current dipole (ECD) models (Stufflebeam et al., 2009; Zhu et al., 2012). ECD models estimate the time-varying locations, orientations, and amplitudes of a small number of (i.e., generally fewer than 10) dipole sources in the brain in order to account for the variance observed in the MEG sensors.

Although ECD modeling helps map sensor activity onto brain structures, it has a limited ability to localize multiple distributed or extended sources, such as those likely recruited in complex cognitive tasks. However, other localization methods have been developed for analyzing MEG data to address some of these limitations. Since time-series analysis of ERF/Ps and ECDs has predominantly received focus in previous work, we will focus here on describing some of these methodological developments in MEG inverse imaging and analysis beyond sensor-space (ERF/P) or ECD modeling of time series, and discuss the potential advantages these methods could confer for clinical use.

Localizing the neural generators in the brain underlying MEG (and/or EEG) measurements is a mathematically under-constrained problem, as there are infinite solutions that could give rise to the observed electro-magnetic patterns (an observation made as early as Helmholtz, 1853). Therefore, any analysis method – including analyzing signals directly from individual sensors or ECD fitting – relies on some set of assumptions to interpret MEG data. Accurately projecting these data onto neural sources in the brain is known as the “inverse problem.” The classic ECD approach, for example, solves this problem by assuming that measurements should be explained as fully as possible by a handful of predefined focal brain sources. By contrast, inverse imaging approaches use a large, fixed set (often thousands) of dipole-like brain sources constructed *a priori* to form what is known as a *source space*, and the time-varying amplitude of each source is estimated to account for the observed sensor data (see Baillet, 2010 for an in-depth discussion). These brain sources are often constrained (assumed) to be approximately normal to the cortical surface using accurate individualized structural MRI information (Dale and Sereno, 1993; Lin et al., 2006), reflecting the sensitivity of MEG predominantly to cortical sources (Hämäläinen et al., 1993). Thus, inverse imaging provides a view into brain activation across all of neocortex, which critically allows for interpretation of the observed activations in terms of underlying brain function. This shift in analysis and interpretation in turn facilitates immediate comparison to and integration with other neuroimaging techniques that image neural activation in the brain (e.g., fMRI or PET) as well as animal models by way of homologous or analogous regions. It also allows us to more readily formulate and test hypotheses regarding the mapping between brain anatomy and cortical (dys)function.

The end result of MEG inverse imaging computations is a set of time-varying estimates of neural activity mapped onto the brain. However, different inverse solutions can be employed based on the task and neural activity under investigation. The minimum-norm estimate (MNE) approach is perhaps the most prominent distributed inverse imaging method (Hämäläinen and Ilmoniemi, 1994). In MNE, neural currents in the brain are assumed to be low-amplitude and distributed broadly, which can be mathematically expressed as minimizing the L2-norm (also known as a Euclidean norm, i.e., the sum of squared values) of currents in the brain. For complex cognitive tasks that are likely to recruit multiple brain regions, MNE is often a good choice because it is designed to localize distributed sources of neural activity. For example, MNE analysis would likely be useful for studying the neural activation during complex auditory or audio-visual attention tasks, since such tasks have been shown to engage broad, distributed cortical networks (e.g., Corbetta et al., 2008). Despite the fact that MNE by definition will create point spread even for focal activations, MNE has been shown to localize focal activity well enough for potential use in clinical applications (Shiraishi et al., 2013). Other inverse methods (e.g., minimum-current or mixed “L1–L2” norm estimates; Matsuura and Okabe, 1995; Uutela et al., 1999; Ou et al., 2009; Gramfort et al., 2012) have been designed specifically for tasks where a limited number of focal activations are expected *a priori* (e.g., N100 localizations that are restricted to primary sensory areas). However, we suggest here that MNE is the best all-around choice for exploring the cortical dynamics in auditory tasks, where potentially extensive cortical networks could be involved (e.g., Larson and Lee, 2013, 2014).

With these time-varying neural activations obtained using inverse imaging, it can be informative to use time-series analyses to identify peak locations (spatio-temporal) regions of significant activation, or latency differences between areas or conditions. Although such time-series analyses have received the largest focus in past studies, a rapidly growing area of research in neuroimaging is in connectivity analysis, which seeks to determine the relationships between two or more neural populations (Friston and Frith, 1995; Banerjee et al., 2012). One advantage of connectivity analysis using MEG is that the fine time resolution allows for analysis of rapidly varying neural activity, compared to fMRI-based connectivity measures, which must operate on the time scale of seconds due to acquisition and physiological constraints (Fox and Raichle, 2007). However, calculating functional connectivity using MEG often requires careful treatment due to signal mixing, point spread, and potentially ambiguous localization. Multiple connectivity measures have thus been recently devised to help compensate for these potential confounds (Vinck et al., 2011), including some that estimate the time-delay in information propagation between sources (Nolte et al., 2008). These types of methods can give us additional insight into how neural structures communicate at the systems-level in order to accomplish different tasks.

## Potential Applications for (C)APD (and Beyond)

We propose that using these MEG analysis methods in concert with targeted behavioral experiments could have a potential impact on the evaluation of pathologies that often manifest during development. Although many such potential applications exist, here we focus on how recently developed MEG methods could be used for diagnosis or therapeutic intervention in (C)APD. Note that although (C)APD is most often diagnosed in children, most of the ideas presented here apply equally well to experiments that involved adults or children with (C)APD.

Listeners with (C)APD often experience difficulties understanding speech in noisy acoustic environments despite having normal audiological pure-tone thresholds. Around 5% of children referred to audiological clinics for listening problems have normal-hearing thresholds, leading to (C)APD diagnoses (Chermak and Musiek, 1997; Moore et al., 2010). Nonetheless, the precise definition of (C)APD is a source of some debate. For example, the American Speech-Language-Hearing Association (ASHA) Technical Report (ASHA Working Group on Auditory Processing Disorders, 2005) describes (C)APD as a deficit in neural processing of auditory stimuli that is not due to higher order language, cognitive, or related factors. However, (central) auditory processing can be defined as broadly as the efficiency and effectiveness by which the central nervous system (CNS) utilizes auditory information, or as narrowly as the perceptual processing of auditory information in the CNS insofar as the neurobiological activity gives rise to electrophysiological auditory potentials.

Many clinicians prefer a related but more functional definition provided by ASHA. In this definition, diagnosis of (C)APD is based on observed difficulties in the perceptual processing of auditory information, as demonstrated by a patient having poor performance in one or more of the following skills: sound localization and lateralization; auditory discrimination; auditory pattern recognition; temporal aspects of audition; or performance with competing acoustic signals and with degraded acoustic signals (ASHA Working Group on Auditory Processing Disorders, 2005). It is further argued that the only tests that can be validly used to diagnose (C)APD are those that can be used to infer a physical lesion in a particular part of the brain (e.g., AAA, 2010). Theoretical models, such as the Buffalo model (Katz, 1992) and the Bellis/Ferre model (Ferre, 1997; Bellis, 2003), have emerged to guide clinicians in their interventions with (C)APD patients by linking some of these behavioral test results with potential dysfunction and/or lesions in the CNS (Jutras et al., 2007). For example, the Buffalo model proposes four (C)APD subcategories, one of which links the ability to understand speech in noise and auditory short-term, working memory, and attention. This is referred to as the “tolerance-fading memory category” implicating a dysfunction in the frontal or anterior-temporal cortex. Similarly, another category (“integration category”) links difficulties integrating auditory and other types of information, such as that from visual stimuli (Stecker, 1998) to expected lesions in the corpus callosum or the angular gyrus (Katz, 1992).

These sorts of theoretical models and their associated lesion studies (Musiek and Sachs, 1980; Bamiou et al., 2007; Boscariol et al., 2010) take important steps in forming a basis for our understanding the role of the CNS in (central) auditory processing. However, requiring or presupposing links between the observed dysfunctions in behavior specifically to brain lesions may be less informative than links to a broader class of neural dysfunction. Here, we believe that modern neuroimaging studies could be used to shed light on these phenomena to identify pathological neural processing during behavior. Moreover, these techniques allow us to carry out hypothesis-driven experiments rooted in theories of cognitive neuroscience to help constrain and test for the neural basis of APD. For example, over the past few decades there has been a growing appreciation that the attentional network in the cortex may be supramodal – that is, auditory and visual attention may utilize shared resources at the cortical level (see Lee et al., 2014 for a review). Neuroimaging can thus help us understand the extent to which supramodal versus auditory-specific attention mechanisms are engaged during complex listening tasks. This positions us in turn to test how neurobiological activity in the cortex is involved in (dys)functional auditory processing. The spatial and temporal resolution of inverse-imaging MEG methods are particularly appropriate for identifying the neural substrates underlying (C)APD. Such a neuroimaging approach could prove useful for (at least) the following four reasons: (1) it would give us insight into the cortical neural mechanisms underlying (C)APD and compensation strategies employed by subjects (thereby complementing ABR analyses that measure sub-cortical mechanisms), (2) it would enable us to link patient’s performance in clinical behavioral tests to these underlying neural structures, (3) it could lead to the development of novel intervention strategies based on the identified neural pathologies, and (4) it would allow for determining the extent to which intervention strategies managed to restore typical neural function or promote compensation strategies.

Recently, a large-scale pediatric study showed that variability in subject responses (as opposed to overall performance levels) during an auditory task better accounted for individual listening ability (Moore et al., 2010). In another study, behavioral tests designed to tax top-down auditory attention were able to account for some of the variability in listening performance in complex settings for normal-hearing adult listeners (e.g., Zhang et al., 2012). These types of results suggest that cognitive factors such as the ability to consciously direct attention to stimuli (or tasks) of interest play an important role in hearing in complex settings. However, the neural underpinnings of such function are only partially understood. For example, we recently found a correlation between how well subjects can voluntarily switch auditory spatial attention and activity in the right temporoparietal junction (Larson and Lee, 2013, 2014), suggesting that an area understood to be involved in visuo-spatial attention (Corbetta et al., 2008) mediates effective control of auditory attention. Whole-brain inverse imaging should help accurately capture and characterize the dynamics of the distributed cortical networks that participate in these tasks, and allow us to form and test hypotheses regarding how attention network dysfunction could manifest in (C)APD.

Further integration of such tasks with MEG recording could allow for informative brain–behavior correlations that lead identification of neural pathologies and/or potential diagnostic strategies for (C)APD. To our knowledge, MEG has not been used to examine the cortical processing involved in (C)APD, perhaps in part because focus has been predominantly on sub-cortical processing in (C)APD. MEG may thus serve as a complement to auditory brainstem response measurements, which have previously been suggested as a potential diagnostic tool (Catts et al., 1996; Chermak and Musiek, 1997), by isolating dynamics at the cortical level that are useful in understanding the relationship between neural processing and behavior in order to aid effective diagnosis via behavioral tests. For example, having subjects with (C)APD perform attention-demanding tasks using auditory and visual stimuli may reveal irregularities in processing or connectivity between the primary sensory areas (i.e., auditory and/or visual cortices) and the cortical attention network. These tasks could thus address the extent to which APD is related to disruptions in modality-specific versus supra-modal network components. In this way, MEG (or even better, combined M/EEG) could be used to extend the findings of previous studies showing differences in cortical processing using EEG (e.g., Kraus et al., 1993; Gavin et al., 2011) by enabling connectivity-based analyses, mapping activity onto neural structures to formulate and test hypotheses regarding the nature of disrupted function, and by providing a complementary view of cortical neural activity that may be involved in (C)APD.

As the co-morbidity of (C)APD with other disorders (such as attention deficit hyperactivity disorder or autism spectrum disorder) may complicate behavioral diagnosis of (C)APD, MEG may prove useful as a tool to help isolate the extent to which different neural structures are involved in each of these pathologies. Other measurements such as ABR would also serve as a useful complement to MEG by measuring the function of midbrain and earlier auditory structures. This would provide a more complete perspective on how the brain may influence subject behavior (e.g., Kraus, 2012), especially in terms of top-down influences from cortical areas (Lehmann and Schönwiesner, 2014). Once MEG is used to isolate specific cortical structures involved in (C)APD, understanding the geometry of the anatomical region involved could facilitate development of an EEG protocol to provide a simpler, easier, and cheaper method to deploy in the clinic. These ideas regarding how MEG could shed light on (C)APD could readily be extended to other pathologies that manifest during development with auditory dysfunction, or to help tease apart the extent to which normal auditory neural function has been disrupted.

## Conclusion

There are multiple strengths of MEG as a neurophysiological measure that have yet to be fully leveraged for evaluating pathologies that manifest during development. Combining improved source localization and connectivity measures with auditory paradigms have increased our capability to identify how neural activity relates to behavior. Given the ability of MEG to track the rapid dynamics that may underlie many forms of normal and abnormal auditory processing, researchers may be able to use MEG as a tool to help understand and diagnose (C)APD, and eventually evaluate the neural outcomes of various treatments.

## Conflict of Interest Statement

The authors declare that the research was conducted in the absence of any commercial or financial relationships that could be construed as a potential conflict of interest.
